# Characterization of dip effect on strength for gently inclined rock pillar

**DOI:** 10.1038/s41598-025-09819-w

**Published:** 2025-07-14

**Authors:** Lijun Sun, Pengcheng Li, Shujian Li, Menglai Wang, Lingpan Du

**Affiliations:** 1 National Engineering Research Center of Phosphorus Resources Development and Utilization, 430073 Yunnan province, China; 2 Sinoeteel Maanshan General Institute of Mining Research CO., LTD, 243000 Anhui Province, China

**Keywords:** Rock pillar, Strength, Dip effect, Compression-shear strength model, Civil engineering, Mechanical engineering, Mineralogy

## Abstract

In underground mining operations, rock pillars play a crucial role as load-bearing elements whose structural integrity exhibits strong correlation with the inclination angle of the ore deposit. While the dip effect on pillar strength is widely acknowledged, quantifying this effect remains challenging. This study addresses this issue through theoretical and numerical approaches. A failure criterion for inclined rock was applied to establish the relationship between flat and inclined rock pillar strength. A dimensionless compression-shear coefficient (incorporating in-situ stress factors) was introduced to bridge this relationship, enabling the development of a mathematical model for estimating pillar strength based on the ore-body dip angle. This model integrates rock strength criteria with empirical formulas, extending the application of rock strength theory. The model’s results were validated against numerical simulations, showing strong agreement. Both methods demonstrated that pillar strength decreases as the dip angle increases. The compression-shear coefficient effectively quantifies the dip effect, revealing a consistent decline in strength with higher dip angles. This research not only provides a theoretical framework for assessing inclined pillar strength but also enhances the practical application of rock strength theory in geomechanical applications.

## Introduction

Many ore raw materials openings always involve leaving portions of the ore as temporary or permanent supporting pillars for underground deposits. For instance, systematically arranged residual ore blocks act as natural stress buffers, providing continuous counterforce to overlying strata while maintaining geometric integrity of underground openings^1^. As an essential supporting structure, the pillar becomes a place of stress concentration. The optimization of pillar geometry parameters must adhere to the dual-objective synergy criterion of strength and economy. The fundamental contradiction lies in achieving maximal load-bearing efficiency of support units while minimizing resource depletion through constitutive modeling, under the precondition of maintaining surrounding rock system stability^2^. Therefore, the estimation of pillar strength has always been an important issue.

**Fig. 1:**
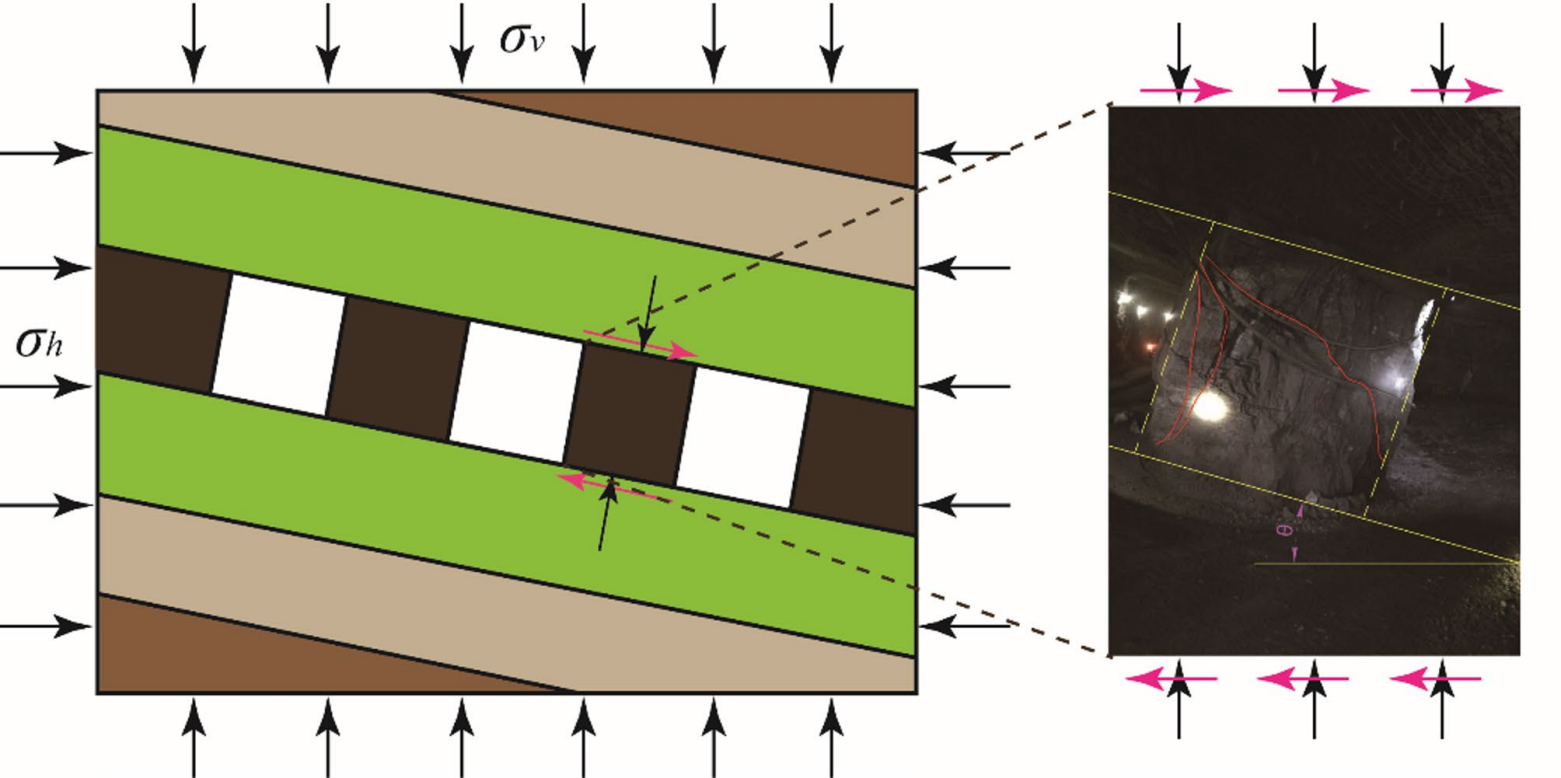
Shear failure of an inclined pillar under combined compression-shear loading.

Affected by the inclination of the orebody, the surrounding rock behavior of inclined orebody mining is different from that of horizontal orebody^3,4^. Inclined pillars are subjected to both compressive and shear load, and they appear in pairs^5^. Under the combined compression-shear load, the shear failure occurs from pillar skin to core, as shown in Fig. 1. Research shown that the additional shear stresses result in significant loss of confinement in the pillar, negatively affecting the load capacity of the rock pillar^6^, which reduces the safety factors^7^, even pillar bursts occur in the same area^8^^[–9^. Thus, the effect of inclination on the pillar strength cannot be ignored in the inclined deposit.

Gently inclined pillars experience combined compression-shear loading due to the angled influence of overburden pressure and associated stress. The rock test is carried out with the same loading path as the inclined pillar, which is the basis of understanding the behavior of the inclined pillar. Based on the properties of compression and shear loading, three distinct test systems have been designed to conduct combined compression-shear experiments on intact rock. The first type of test system was developed by Dr. Fidelis Suorinenin^10^, this system was employed to study mechanical properties of basalt, granite, and coal specimens at various inclination angles^10–13^, the results show that specimen inclination has remarkable effects on the microcrack initiation, microcrack damage and ultimate failure modes of the basalt/granite/coal specimen. The failure patterns of samples are inevitably converted to shear failure from axial splitting with increasing specimen dip angle, and the peak strength of samples decreases as the specimen inclination increases^10–13^. The second type of test system was created by shaping the samples with inclined planes at both ends, enabling the combined compression-shear testing of the specimens^14^–^15^. The third type of test system was developed by adding a double-sided symmetrical cushion block on the universal material testing machine, but two samples need prepare for each test^16–18^. The three types of test systems mentioned above can all realize the combined compression-shear loading and obtain the behavior and strength characteristics of the rock. Studies indicate a significant relationship between the strength of rock specimens subjected to combined compression-shear loading and their strength under uniaxial compression. This correlation was presented in the form of an equation using the method of mathematical statistics^11,13,19^. In addition, this correlation was also successfully expressed with a formula based on Mohr-Coulomb criterion^18^. Though the correlation between rock specimen strength and specimen inclination has been built, this relationship has not been used to characterize the dip effect on pillar strength.

**Fig. 2:**
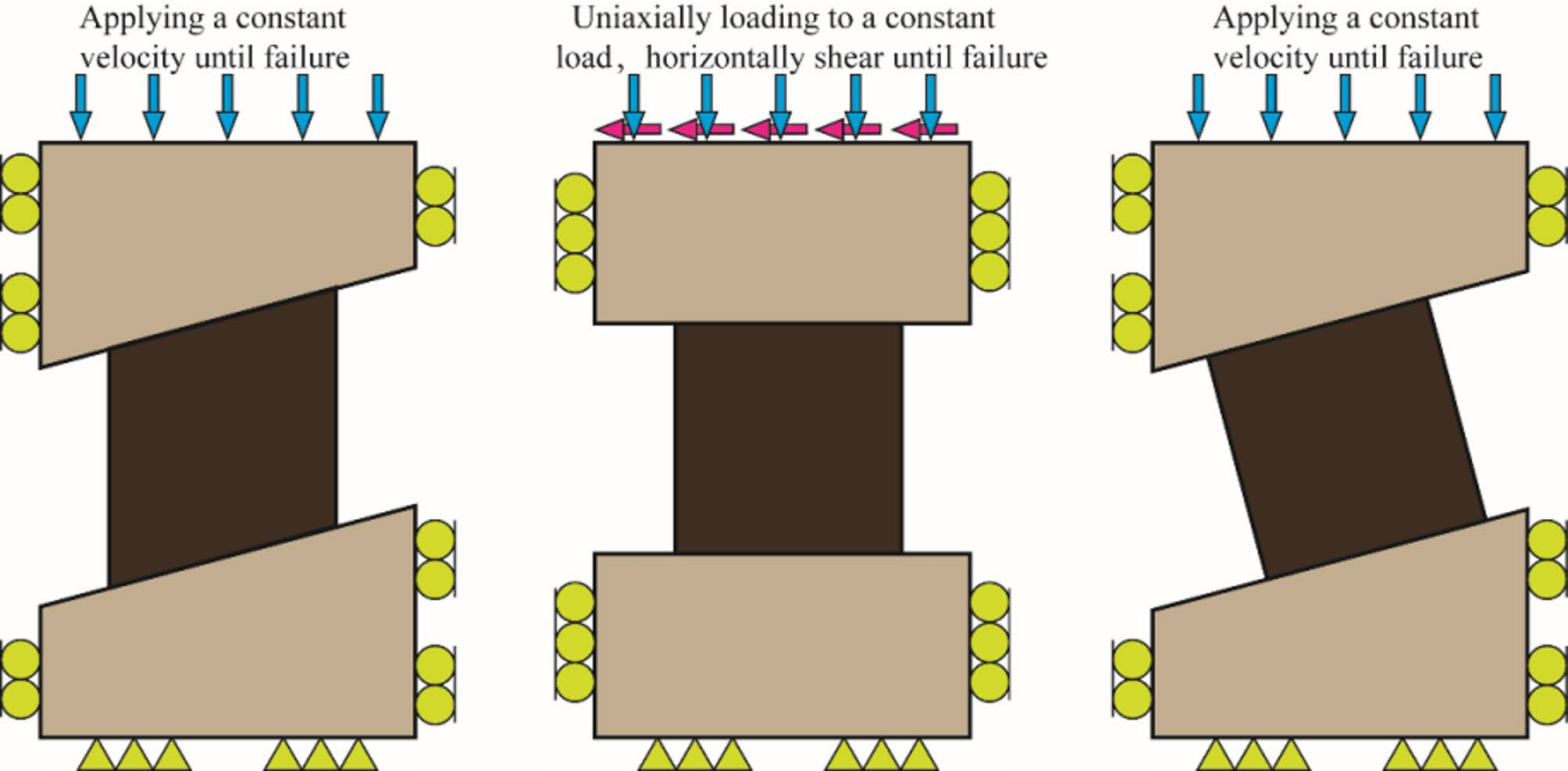
Simplified pillar geometry; (**a**) type-1 geometry; (**b**) type-2 geometry; (**c**) type-3 geometry.

Numerical modeling is one of the effective methods to gain rock pillar strength, as long as parameters are calibrated. Obtaining the flat rock pillar strength has been a mature technology using numerical methods^20–22^. In recent years, numerical modeling has been employed to study the behavior and strength of inclined pillars. Based on the compression-shear load properties of these pillars, researchers have simplified three types of pillar geometry to explore the connection between strength and dip angle, as illustrated in Fig. 2. Type-1 is that the sidewalls of the pillars were assumed to be vertical^23–26^, as shown in Fig. 2a. In type-2, a flat pillar model was created, and a constant uniform velocity is applied to the overlying strata of the pillar, horizontally shear the pillar until failure occurred to compute the pillar strength^6^, as shown in Fig. 2b. For type-3, the sidewalls of pillars were assumed to be perpendicular to the overlying and the underlying strata of the pillar^27–30^, as shown in Fig. 2c. Results from the numerical modeling indicate that the stress of the horizontal pillar is symmetric, while the inclined pillar is asymmetrical. Dipping pillars have reduced strength compared with horizontal pillars, and lower strength results from steeper inclinations. Studies reveal that, when an interface between the pillar and the rock stratum is taken into account, the average pillar strength of the type-3 geometry surpasses that of the type-1 geometry. However, in the absence of interfaces, the type-1 geometry exhibits greater strength than the type-3 geometry^31^. As the research mentioned above, there is no doubt that the dip angle has a great influence on the pillar strength, which has become a generally recognized viewpoint. At present, characterizes the inclination effect on pillar strength is an important work using a mathematical expression. A little work has already been done, on the one hand, from numerical results the dip effect on pillar strength was characterized by the statistical method of the multivariate non-linear regression^25^. On the other hand, the relationship between the strength of the inclined rock and the uniaxial compressive strength is established by fitting the experimental data, which was used to modify the empirical formula for flat pillar strength^13^. However, though some efforts have been an attempt, developing a mathematical model or formula to characterize the dip effect on pillar strength is still a difficult task.

The objective of this research is to develop a model to characterize the dip effect on pillar strength and provide a theoretical basis for estimating the strength of inclined rock pillars. Based on the failure criterion of inclined rock, the correlation between flat pillar and inclined pillar strength was derived through a dimensionless compression-shear coefficient incorporating in-situ stress factors. A strength model incorporating dip angle and the compression-shear coefficient was developed. The pillar strength calculated by the model was compared with that obtained from numerical simulations, showing strong agreement, and the parameters of the developed model were systematically analyzed to quantify the dip effect.

## Development of analytical model for strength of inclined rock pillar

**Fig. 3:**
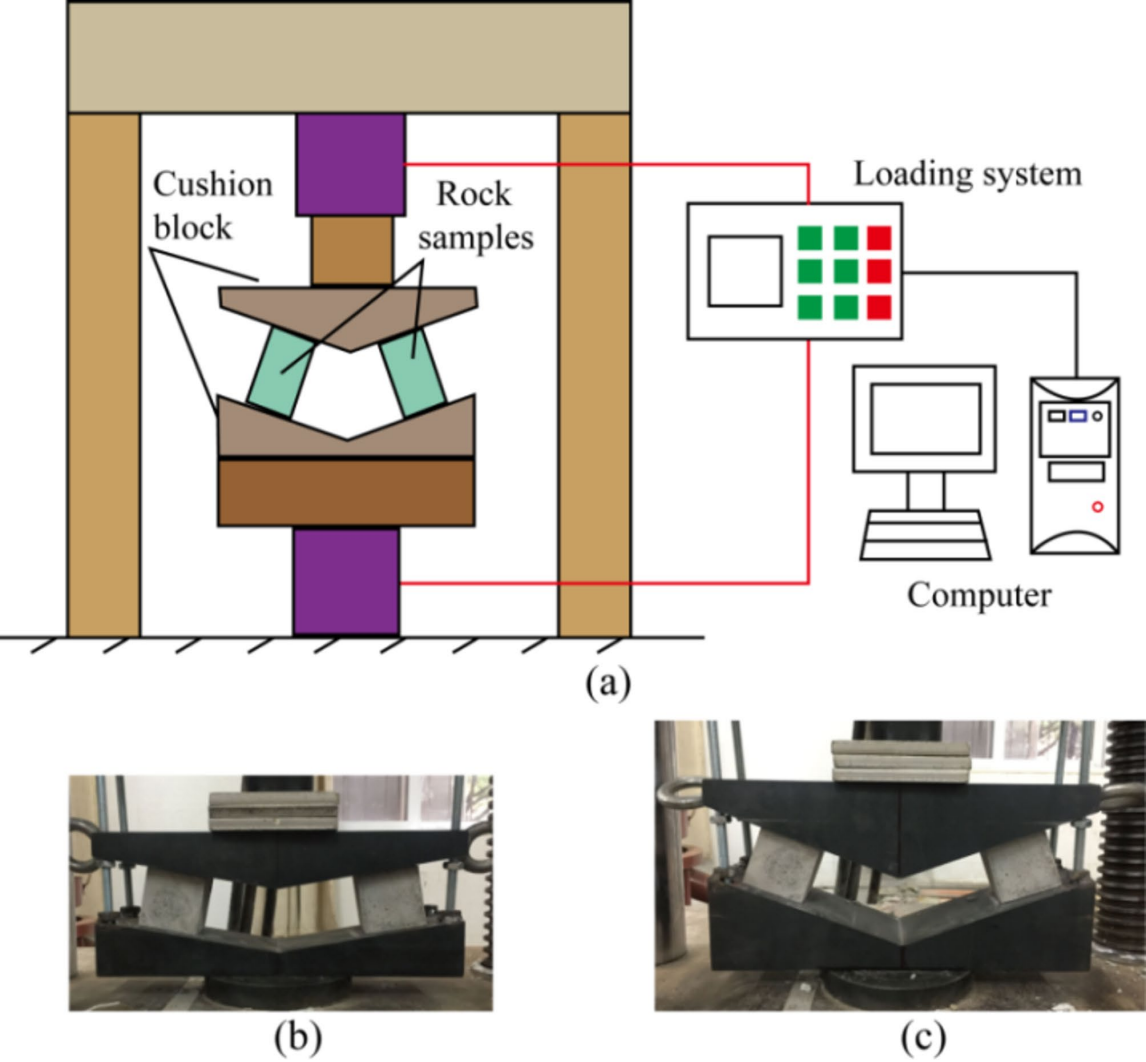
Compression-shear loading test system and cushion block (Luo et al^18^.); (**a**) Compression-shear loading test system; (**b**) Dip of sample with 10°; (**c**) Dip of sample with 20°.

The inclined pillar experiences combined compression-shear loading. Laboratory tests of rock under such loading conditions provide the foundation for understanding the inclined pillar’s behavior and are crucial for scaling experimental data to field applications. In previous research, a set of test devices has been developed to study the mechanical behavior and strength characteristics of rock under combined compression-shear load, as shown in Fig. 3. Combined with the test data, the failure criterion of rock was proposed under combined compression-shear loading^18^.

The following assumptions are made in this study:

1. The influence of cracks and joints is negligible, and the elastic phase conforms to the fundamental assumptions of elasticity theory;
2. The research focuses on hard rock pillars, and interface problems that may arise in weak interlayers are not considered.

**Fig. 4:**
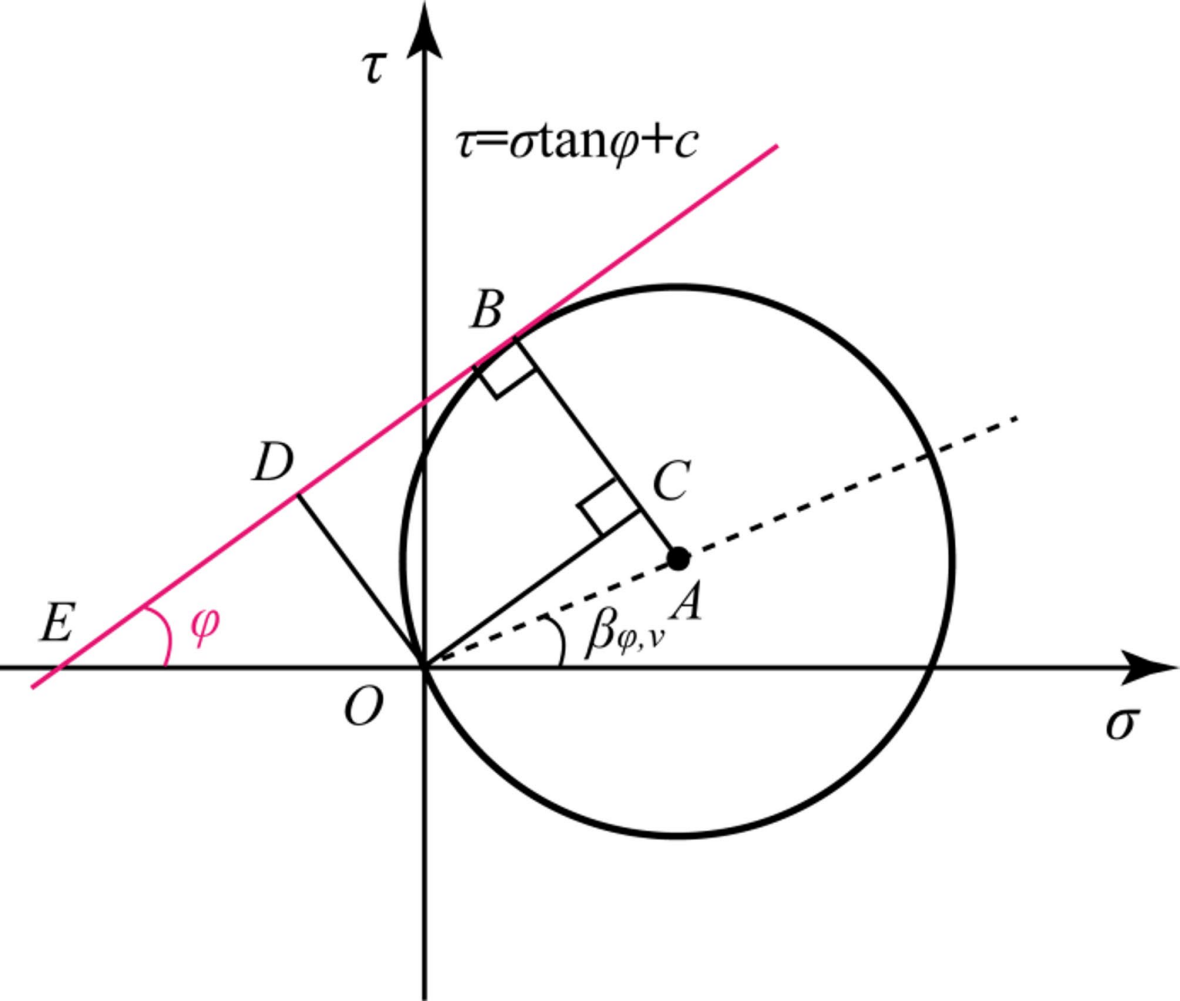
The relationship between stress circle and strength envelope^18^.

The relationship between the ultimate stress circle of rock and the Mohr-Coulomb envelope is characterized according to the compressive-shear characteristics of rock, as shown in Fig. 4. Subsequently, the stress limit equilibrium state equation was formulated as follows.

1 $$\sqrt {{\sigma _\theta }^{2}+{\tau _\theta }^{2}} =\frac{{2c\cos \varphi }}{{1 - \sin \left( {\varphi - {\beta _{\theta,v}}} \right)}}$$

Where *φ* and *c* are the internal friction angle and cohesion of the rock sample, respectively; *β*_*θ, ν*_ is called the effective friction angle of the rock, which is the dip of a straight line on which the center of the generalized Mohr’s circle is located:

2 $$\tan {\beta _{\theta,v}}=\frac{{{\tau _\theta }}}{{{\sigma _\theta }}}=\frac{{\tan \theta }}{{2\left( {1+v} \right)}}$$

Where *θ* is the inclination of the rock sample; *ν* is Poisson’s ratio.

In Eq. (1), the left-hand side represents the actual stress experienced by the rock samples. If this value is less than the right-hand side, the rock sample remains stable; otherwise, it will fail. By integrating Eqs. (1) and (2), the strength of the sample, denoted as σ_θc_, is determined under combined compression-shear loading conditions.

3 $${\sigma _{\theta c}}=\frac{{2c\cos \varphi }}{{1 - \sin \left( {\varphi - \arctan \frac{{\tan \theta }}{{2(1+v)}}} \right)}}$$

Furthermore, the uniaxial compressive strength of rock can be determined using the Mohr-Coulomb strength criterion:

4 $${\sigma _c}=\frac{{2c\cos \varphi }}{{1 - \sin \varphi }}$$

Combined Eqs. (3) and (4), a dimensionless coefficient f(φ, β_θ, ν_), named compression-shear coefficient, is equal to the ratio of the strength of rock under uniaxial compression to that under the combined compression-shear loading, which was introduced to characterize the dip effect on rock strength.

5 $$f(\varphi,{\beta _{\theta,v}})=\frac{{1 - \sin \varphi }}{{1 - \sin \left( {\varphi - \arctan \frac{{\tan \theta }}{{2(1+v)}}} \right)}}$$

Equation (5) indicates that the compression-shear coefficient f(φ, β_θ, ν_) depends on the internal friction angle *φ*, the sample’s inclination angle and Poisson’s ratio *ν*. Additionally, the inclined uniaxial compressive strength is calculated as the product of the uniaxial compressive strength and the compression-shear coefficient^18^.,

6 $${\sigma _{\theta c}}=f(\varphi,{\beta _{\theta,v}}){\sigma _c}=\frac{{1 - \sin \varphi }}{{1 - \sin \left( {\varphi - \arctan \frac{{\tan \theta }}{{2(1+v)}}} \right)}}{\sigma _c}$$

Equation (6) represents the strength criterion for inclined rock, derived from the Mohr-Coulomb criterion.

For pillars in flat layered deposits, the pillar is loading under pure pressure. Compared with the coal pillar, the focus on the flat rock pillar strength is paid more attention to later. After decades of research, many empirical formulas for rock pillar strength have been proposed, which can be summarized in three forms^32^.

Linear pillar strength formula:

7 $${S_{p0}}=k{\sigma _c}\left( {A+B\frac{w}{h}} \right)$$

Where *S*_*p0*_ is the strength of flat rock pillar; k is pillar strength size factor; σc is the uniaxial compressive strength of pillar material; *A* and *B* are dimension parameters, and *A* + *B* = 1; *w* and *h* are the width and height of the rock pillar, respectively.

Power law strength formula:

8 $${S_{p0}}=k{\sigma _c}\frac{{{w^a}}}{{{h^b}}}$$

Where *a* is the exponent describing the influence of pillar width on pillar strength, *b* is the exponent describing the influence of pillar height on pillar strength.

The confinement strength formula:

9 $${S_{p0}}=k{\sigma _c}(C+Dk)$$

Where *k* is pillar strength size factor determined to be 0.44; *C* and *D* are empirical rock mass constants determined to be 0.68 and 0.52, respectively; *κ* is rock pillar friction term, which could be determined as:

10 $$k=\tan \left( {{{\cos }^{ - 1}}\frac{{1 - {C_{pav}}}}{{1+{C_{pav}}}}} \right)$$

Where *C*_*pav*_ is the average rock pillar confinement, which can be obtained by:

11 $${C_{pav}}=0.46{\left[ {\log \left( {\frac{w}{h}+0.75} \right)} \right]^{\frac{{1.4}}{{(w/h)}}}}$$

**Fig. 5:**
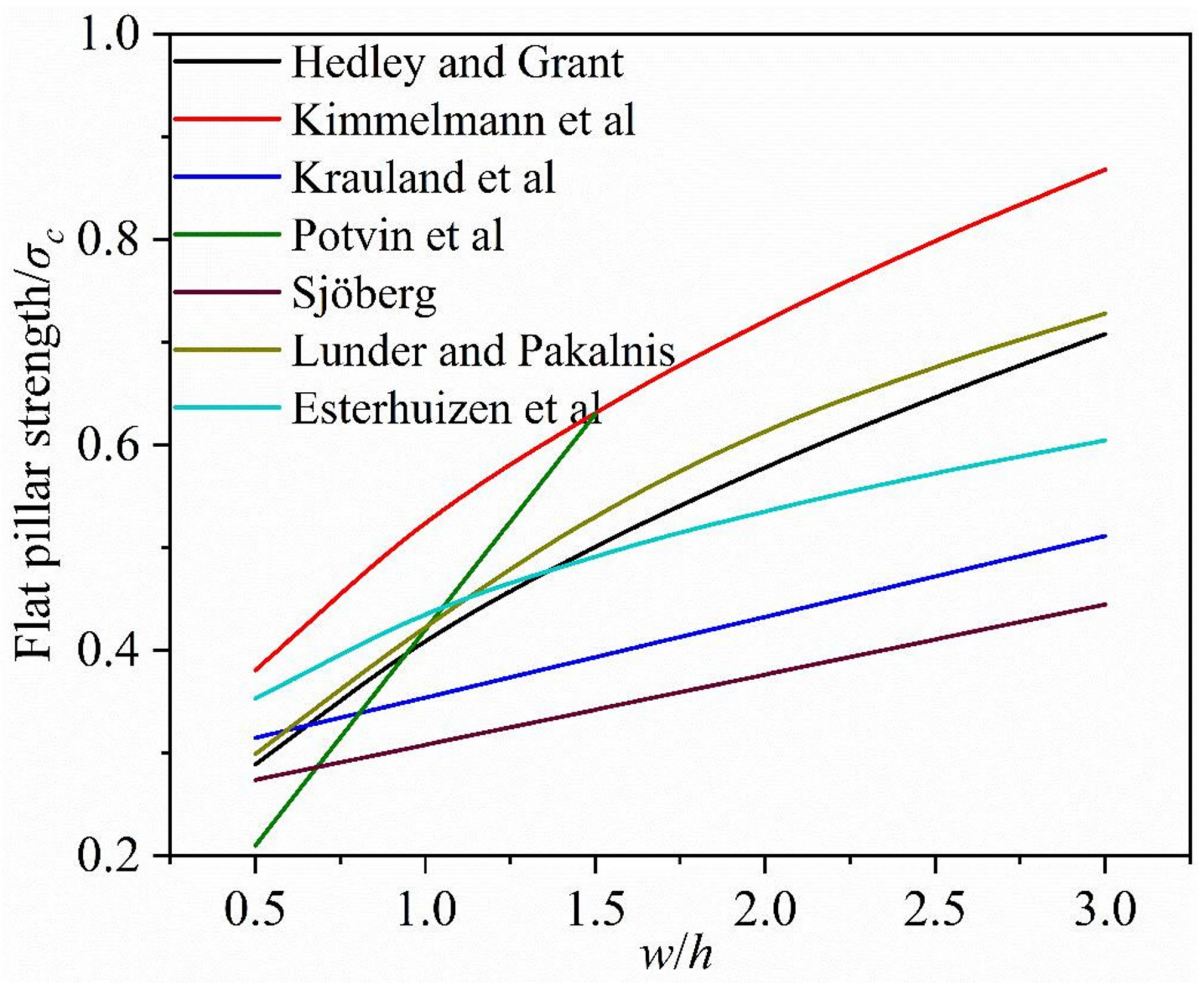
Comparison of several flat rock pillar strength equations.

The abovementioned three forms for details of commonly used strength formulas are given in Table 1. From these formulas, using a pillar height of 4 m, the relationship curves between the normalized strength and width to height ratio are shown in Fig. 5. Due to the different nature of rock pillar materials, different pillar strength formulae have different normalized strength having the same size pillar. Therefore, when the strength formulae are employed to estimate the rock pillar strength, similar material properties of the rock pillar should to be considered.

**Table 1:** Summary of flat rock pillar formulae.

Source	Rock Pillar Strength formula	Form	Remark
Hedley^33^	${S_{p0}}=0.578{\sigma _c}\frac{{{w^{0.5}}}}{{{h^{0.75}}}}$	power law	*σ*_*c*_ = 230 MPa
Kimmelmann^34^	${S_{p0}}=0.691{\sigma _c}\frac{{{w^{0.46}}}}{{{h^{0.66}}}}$	power law	*σ*_*c*_ = 94 MPa
Krauland^35^	${S_{p0}}=0.354{\sigma _c}\left( {0.778+0.222\frac{w}{h}} \right)$	linear	*σ*_*c*_ = 100 MPa
	${S_{p0}}=0.420{\sigma _c}\frac{w}{h}$	power law	-
Potvin^36^			
Sjöberg^37^	${S_{p0}}=0.308{\sigma _c}\left( {0.778+0.222\frac{w}{h}} \right)$	linear	*σ*_*c*_ = 240 MPa
Lunder et al^38^	${S_{p0}}=0.44{\sigma _c}\left( {0.68+0.52\kappa } \right)$ Where$$\left\{ \begin{gathered} \kappa =\tan \left( {{{\cos }^{ - 1}}\frac{{1 - {C_{pav}}}}{{1+{C_{pav}}}}} \right) \hfill \\ {C_{pav}}=0.46{\left[ {\log \left( {\frac{w}{h}+0.75} \right)} \right]^{\frac{{1.4}}{{(w/h)}}}} \hfill \\ \end{gathered} \right.$$	confinement	-
Esterhuizen et al^32^	${S_{p0}}=0.65{\sigma _c} \cdot LDF \cdot \frac{{{w^{0.30}}}}{{{h^{0.59}}}}$	power law	*LDF* represents the coefficient of discontinuity, and its value ranges from 0 to 1.

From Eqs. (7), (8) and (9), the uniaxial compressive strength of rock pillar material is one of the indispensable factors in the flat rock pillar strength formula. The form that flat rock pillar strength Sp0 can be represented by^1^:

12 $${S_{p0}}={S_{p0}}\left( {{\sigma _c},{\text{size}},{\kern 1pt} {\kern 1pt} {\kern 1pt} {\text{shape}}} \right)$$

The inclined rock pillar is under combined compression-shear load, similar to the horizontal rock pillar, the strength of the inclined pillar is also contributed by the compression-shear strength of the pillar material. Therefore, assuming that the form of inclined pillar strength *S*_*pθ*_ can be represented by

13 $${S_{p\theta }}={S_{p\theta }}\left( {{\sigma _{\theta c}},{\text{size}},{\kern 1pt} {\kern 1pt} {\kern 1pt} {\text{shape}}} \right)$$

Where *S*_*pθ*_ represents the strength of inclined pillar; *σ*_*θc*_ represents the strength of pillar materials under combined compression-shear loading.

As mentioned in Table 1, when expressing the Eq. (12), there are three forms and many specific formulas. In all these formulas, the pillar strength equation of Lunder & Pakalnis^38^ is the most recent major development in the empirical study of hard rock pillar strength, and is widely accepted rock pillar strength equation^39^. If the size and shape of the pillar are the same, the difference between Eq. (12) and Eq. (13) is that the strength parameters of the rock, Eq. (12) is characterized by uniaxial compressive strength and Eq. (13) is characterized by compression-shear strength. Then Eq. (13) is expressed in the form of Lunder & Pakalnis into

14 $${S_{p\theta }}=k{\sigma _{\theta c}}\left( {C+D\kappa } \right)$$

Other parameters are kept consistent with the flat pillar strength formulae. Then Eq. (6) is substituted into Eq. (14), and the inclined pillar strength formula is obtained,

15 $${S_{p\theta }}=k\frac{{1 - \sin \varphi }}{{1 - \sin \left( {\varphi - \arctan \frac{{\tan \theta }}{{2\left( {1+\nu } \right)}}} \right)}}{\sigma _c}\left( {C+D\kappa } \right)$$

According to Eq. (15), the strength of an inclined pillar can be calculated as the product of the compression-shear coefficient and the flat pillar strength,

16 $${S_{p\theta }}=f\left( {\varphi,{\beta _{\theta,\nu }}} \right){S_{p0}}{e^{i\theta }}$$

Equation (16) is the expression of the relationship between the strength of inclined and horizontal rock pillars, which shows that the compression-shear coefficient of rock is used to build a bridge between the strength of inclined and flat rock pillars. And successfully connect both inclined and flat rock pillar strength. The strength model of the inclined rock pillar is established successfully.

In fact, after the orebody is mined, the load on the pillar is determined by the in-situ stress state and the recovery ratio. The proposed inclined pillar strength model (Eq. (16)) doesn’t consider the effect of the in-situ stress ratio. Therefore, the proposed strength model needs to be further modified. As the expression of the strength model shows, it is the compression-shear coefficient that needs to modify. The inclined pillar is subjected to compression and shear load, and the average compression and shear load component can be obtained by using the tributary area method^5,40^,

17 $$\left\{ \begin{gathered} {\sigma _{p\theta }}=\frac{{\gamma H\left( {\lambda {{\sin }^2}\theta +{{\cos }^2}\theta } \right)}}{{1 - \eta }} \hfill \\ {\tau _{p\theta }}=\frac{{\left| {\left( {1 - \lambda } \right)} \right|\gamma H\sin \theta \cos \theta }}{{1 - \eta }} \hfill \\ \end{gathered} \right.$$

Where *η* is the area extraction ratio, defined by (area mined)/(total area of orebody; *γ* is the weight of the overlying strata rock mass. *θ* is the dip angle of orebody; *H* is the average buried depth of the orebody. *λ* is the ratio of average horizontal stress to vertical stress, called in-situ stress ratio.

In fact, the effective internal friction angle of the rock pillar *β*_*θ, λ*_ is different from that of the rock. And the effective internal friction angle of the pillar is determined by the compression and shear load on the rock pillar. Similarly, from Eq. (2), the relationship between the effective internal friction angle of the rock pillar and the compression and shear load could be obtained.

18 $$\tan {\beta _{\theta,\lambda }}=\frac{{{\tau _{p\theta }}}}{{{\sigma _{p\theta }}}}=\frac{{\left| {\left( {1 - \lambda } \right)} \right|\sin 2\theta }}{{\left( {1+\lambda } \right)+\left| {\left( {1 - \lambda } \right)} \right|\cos 2\theta }}$$

Therefore, the compression-shear coefficient *f(φ*,* βθ*,* λ)* can be obtained considering the inclination angle and the in-situ stress ratio,

19 $$f\left( {\varphi,{\beta _{\theta,\lambda }}} \right)=\frac{{1 - \sin \varphi }}{{1 - \sin \left( {\varphi - \arctan \frac{{\left| {\left( {1 - \lambda } \right)} \right|\sin 2\theta }}{{\left( {1+\lambda } \right)+\left| {\left( {1 - \lambda } \right)} \right|\cos 2\theta }}} \right)}}$$

Furthermore, the strength model considering both inclination and in-situ stress ratio effect is expressed in the following form

20 $${S_{p\theta }}=f\left( {\varphi,{\beta _{\theta,\lambda }}} \right){S_{p0}}$$

Equation (20) is the modified expression of the relationship between the strength of inclined and flat rock pillar.

## Assessment of inclined pillar strength using numerical method

### Numerical simulation process

#### Deposit background and simulation scheme

A phosphate deposit is located in the west of Hubei Province, China. Phosphate orebody is produced along with the stratum. The dip angle of the main orebody varies from 6° to 15°. The thickness of phosphate rock is from 0.63 m to 7.23 m. The roof and floor are mainly medium-thick layered powdery dolomite. The surrounding rock belongs to the middle hard and brittle rock mass. The minimum buried depth of the borehole orebody is equal to 243 m, while the maximum buried depth is equal to 935 m.

**Fig. 6:**
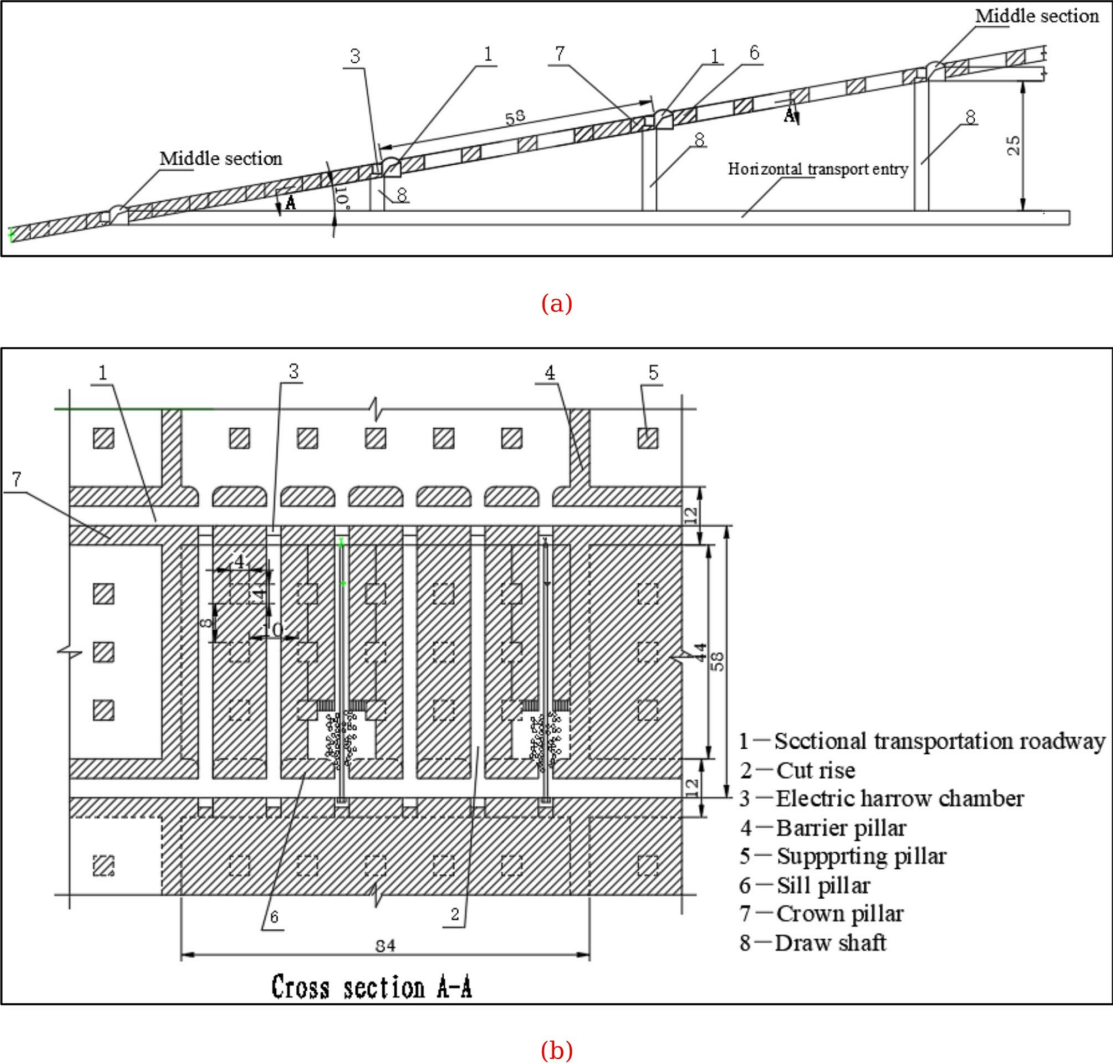
Pillar layout for extraction of an inclined phosphate orebody; (**a**) Section view; (**b**) Top view.

The phosphate orebody mining process was designed using the room-and-pillar method. The space distance between the two pillars is 10 m in strike direction and 8 m in dip direction. The pillar sizes were designed with a length and width of 4 m × 4 m, and the height of the pillar is determined according to the change of the height of the orebody. The pillar and stope layout for extraction of an inclined phosphate orebody are shown in Fig. 6(The length is measured in meters (m)). Most of the orebodies are extracted, while portions remain as support pillars.

**Fig. 7:**
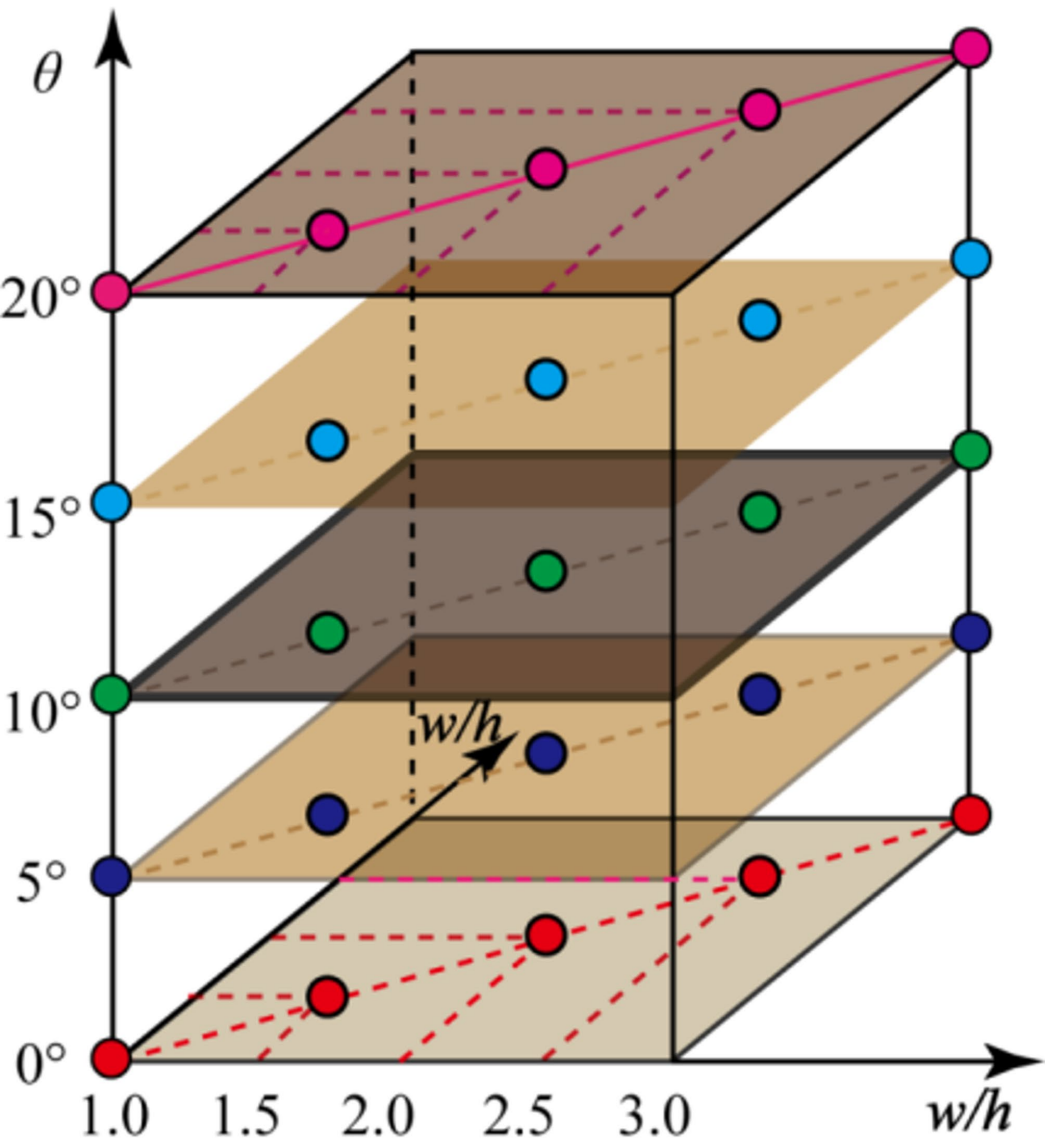
Numerical simulation schemes of pillar strength.

To study the influence of dip angle and width-to-height ratio on pillar strength, according to the occurrence conditions of the orebody, five kinds of orebody dip angle and five types of pillar width-to-height ratio need to prepare. A total of 25 schemes need prepare, as shown in Fig. 7. And the five dip angles can be regarded as five levels at which the strength of pillars with different width-to-height ratios can be evaluated.

#### Numerical model and boundary conditions

**Fig. 8:**
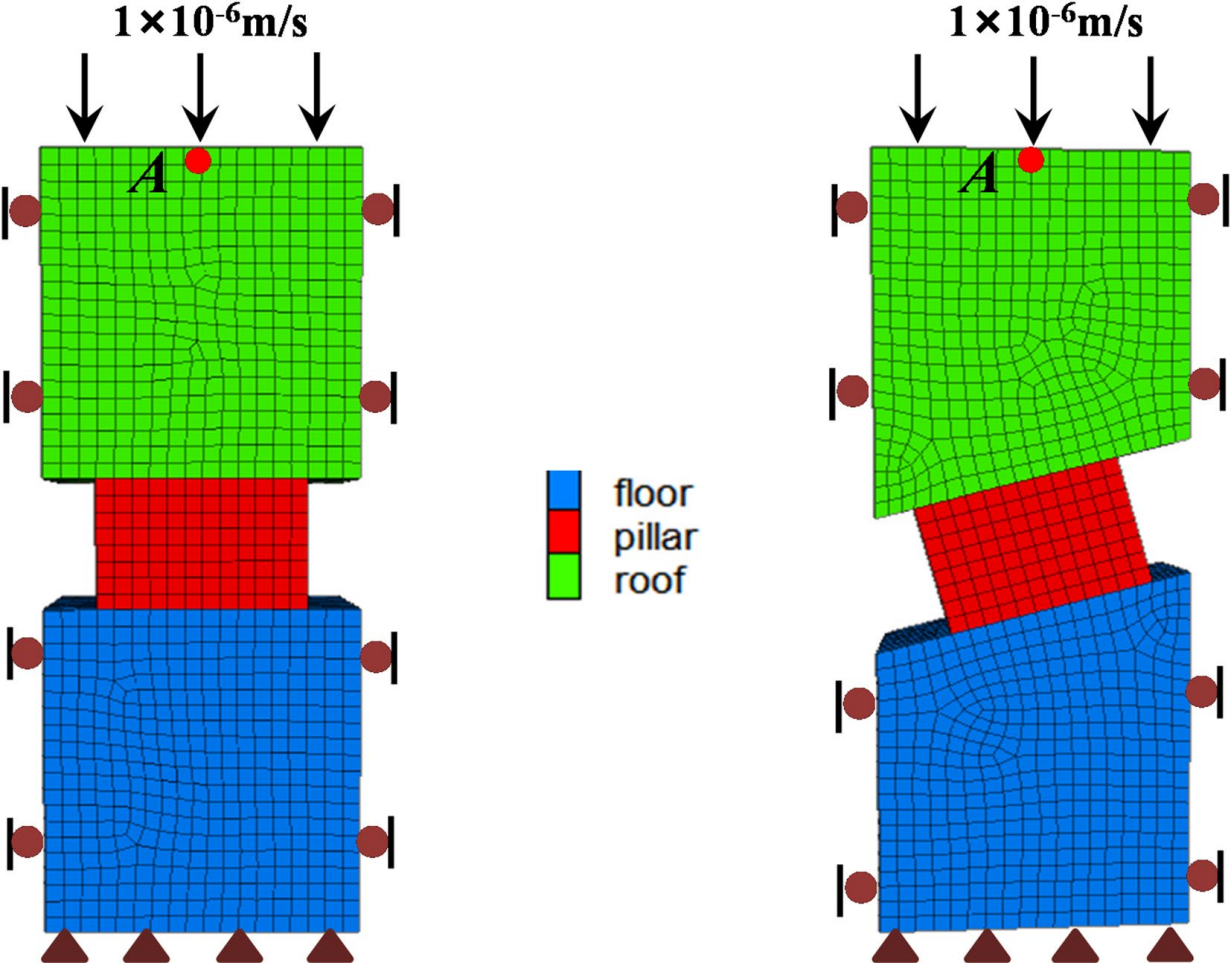
Pillar models (w/h = 1.5); (**a**) Flat pillar; (**b**) Inclined pillar.

Numerical modeling has been a very effective approach, which is widely employed to evaluate pillar strength. The FLAC^3D^ software (Fast Lagrangian Analysis of Continua in 3-Dimensions)^41^ is employed for the assessment of inclined as well as flat phosphate pillar strength. Pillar models were generated at width-to-height ratios of 1.0, 1.5, 2.0, 2.5, and 3.0. Pillar height is fixed to 4 m. Considering the symmetry of the pillar model, half of the pillar model can build as shown in Fig. 8. The zone size of the pillar is kept constant as 0.5 m × 0.5 m × 0.5 m.

**Fig. 9:**
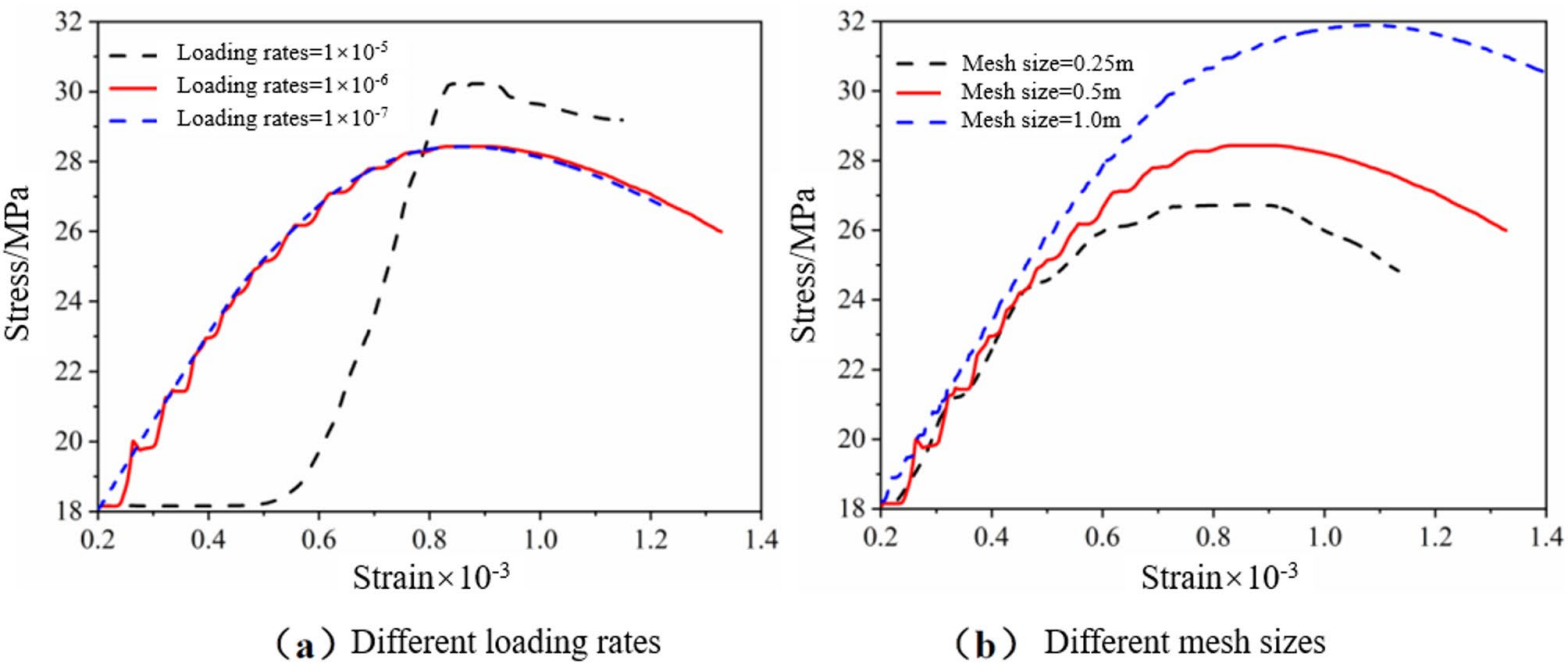
The influence of loading rate and mesh size on the stress-strain curve.

The strain-softening model is applied for a rock pillar, which is based on the Mohr-Coulomb model, and the roof and floor are selected using the Mohr-Coulomb model. The displacement of four vertical symmetry planes of the model is restricted in the normal direction, and zero vertical displacements are set at the base of the model. The models were run to equilibrium under elastic conditions subject to vertical field stress of 8.1 MPa, simulating the orebody at 300 m depth. Both horizontal stresses were set to 6.48 MPa. The in-situ stress ratio is taken to be about 0.8. FLAC^3D^ is commonly used to simulate the loading response mechanisms and strength characteristics of pillars, where the mesh size of the pillar and the loading rate significantly influence the results. In previous numerical simulation studies of pillars, the mesh size typically ranged from 0 m to 1 m, and the loading rate was set between 1 × 10^−7^ m/step and 1 × 10^−5^ m/step. The stress-strain relationship of the pillar is shown in Fig. 9. A loading rate of 1 × 10⁻⁶ m/s was selected and applied uniformly to the top of the model to generate vertical loading. Additionally, a mesh size of 0.5 m was chosen for the numerical simulations. And the displacement of point A was monitored at the top of the model. The load-displacement relation curve was obtained by using the internal programming language available in FLAC^3D^ software. Then a single pillar is modeling under different inclination angles that varied from 0° to 20° at 5° increments.

#### Determination of model parameters

**Fig. 10:**
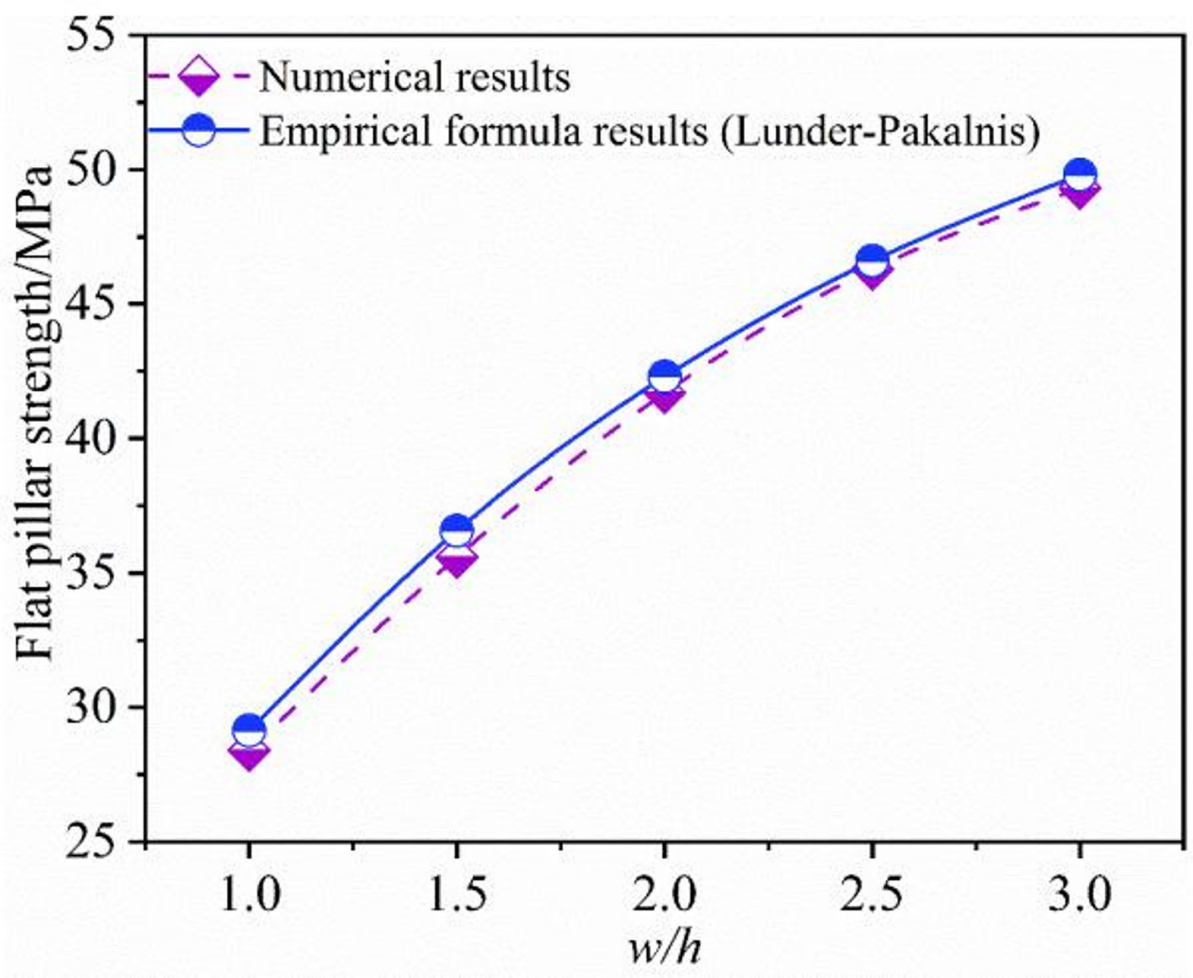
Calibration chart for numerical models and empirical formula.

In this research, the confinement formula^38^ (Eq. (9)) was used to calibrate the model’s material parameter for width-to-height ratios with 1.0, 1.5, 2.0, 2.5, and 3.0 for flat phosphate pillars. The final calibration results give a phosphate rock uniaxial compressive strength of 24 MPa. Tensile strength is set to 10% of the uniaxial compressive strength. The strength of intact phosphate rock is equal to 69 MPa, which was obtained from laboratory tests. Previous studies have shown that the uniaxial compressive strength of rock mass is 0.3–0.5 times those of the intact rock^22^. Thus, the result of parameter calibration meets this case. The pillar material shows 2% shear deformation, the cohesion decreases, and the internal friction angle changes by 2 ~ 3°. The final mechanical parameters are listed in Tables 2 and 3. The rock pillars strength obtained by the numerical method was compared to the empirical formula results shown in Fig. 10. The numerical results are consistent with the empirical formula results, and there is little difference in the strength value calculated^42^. On this basis, the strength of the inclined pillar is obtained from the numerical model.

**Table 2:** Mechanical parameters for numerical model.

Rock mass	Uniaxial compressive strength/MPa	Tensile strength/MPa	Elastic modulus/GPa	Friction/°	Cohesion/MPa	Poisson’s ratio
Rock pillar	24.4	2.30	38.40	32.00	6.24	0.18
Roof/Floor	44.5	4.40	41.50	42.62	12.12	0.22

**Table 3:** Input parameters for strain-softening models.

Plastic shear strain	Cohesion/MPa	Friction/°
0.00	6.40	31.8
0.01	0.80	28.8
0.02	0	26.8
0.50	0	26.8

### Numerical results

#### Load-displacement curve of pillar base

**Fig. 11:**
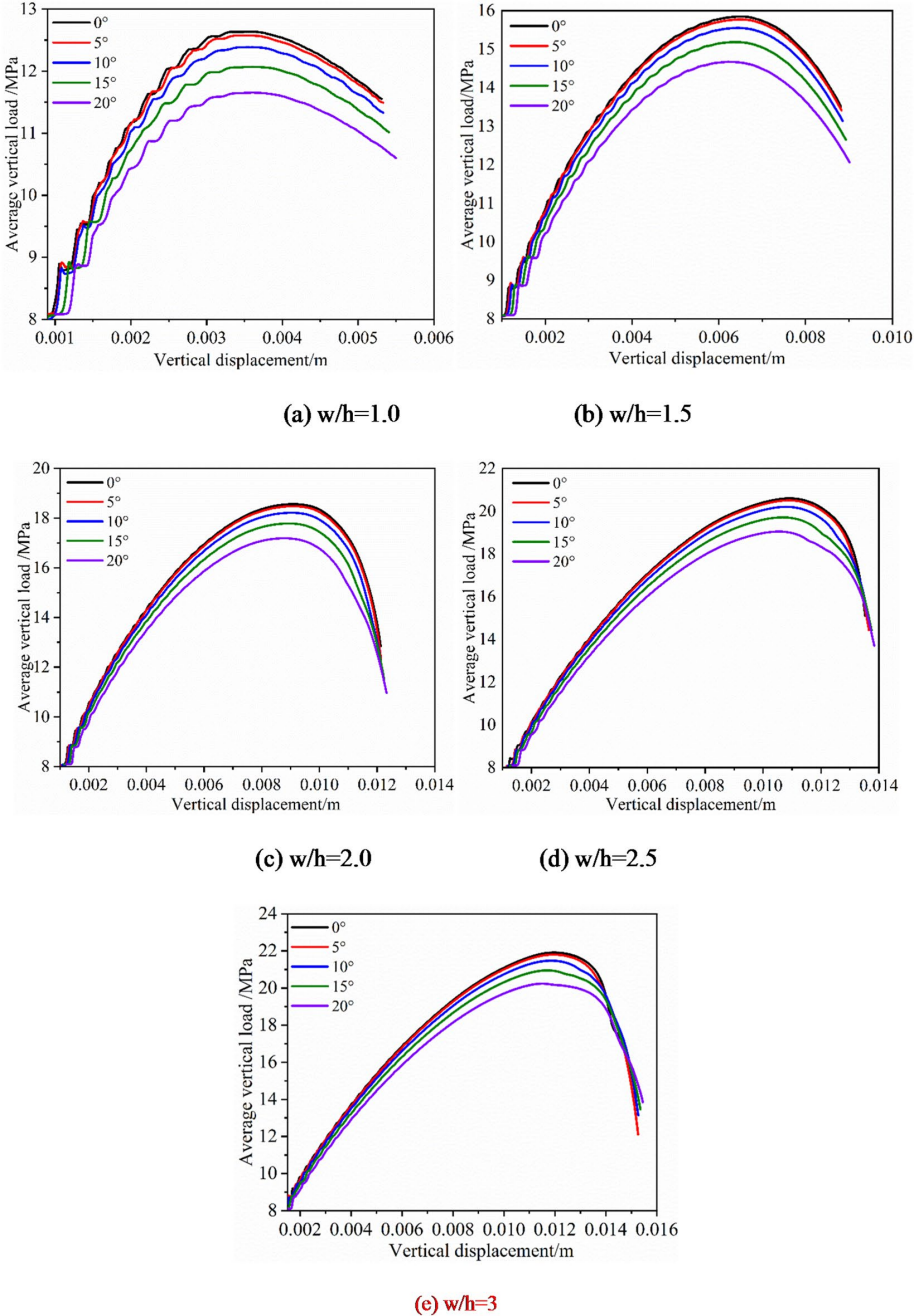
Average vertical stresses versus vertical displacements on the base of pillars.

The average vertical load of the pillar base was calculated by using the internal programming language until the pillar yield and fail. The peak load represents the maximum bearing capacity of the pillar. At the same time, vertical displacement was recorded as well, so that base load-displacement curves could be plotted, as shown in Fig. 11. The variation law of these curves is similar to the stress-strain curves of rock under the uniaxial compression test from the laboratory. When the width-to-height ratio is constant, the load of pillar failure decreases with the increasing inclination angle.

#### Peak stress characteristics

**Fig. 12:**
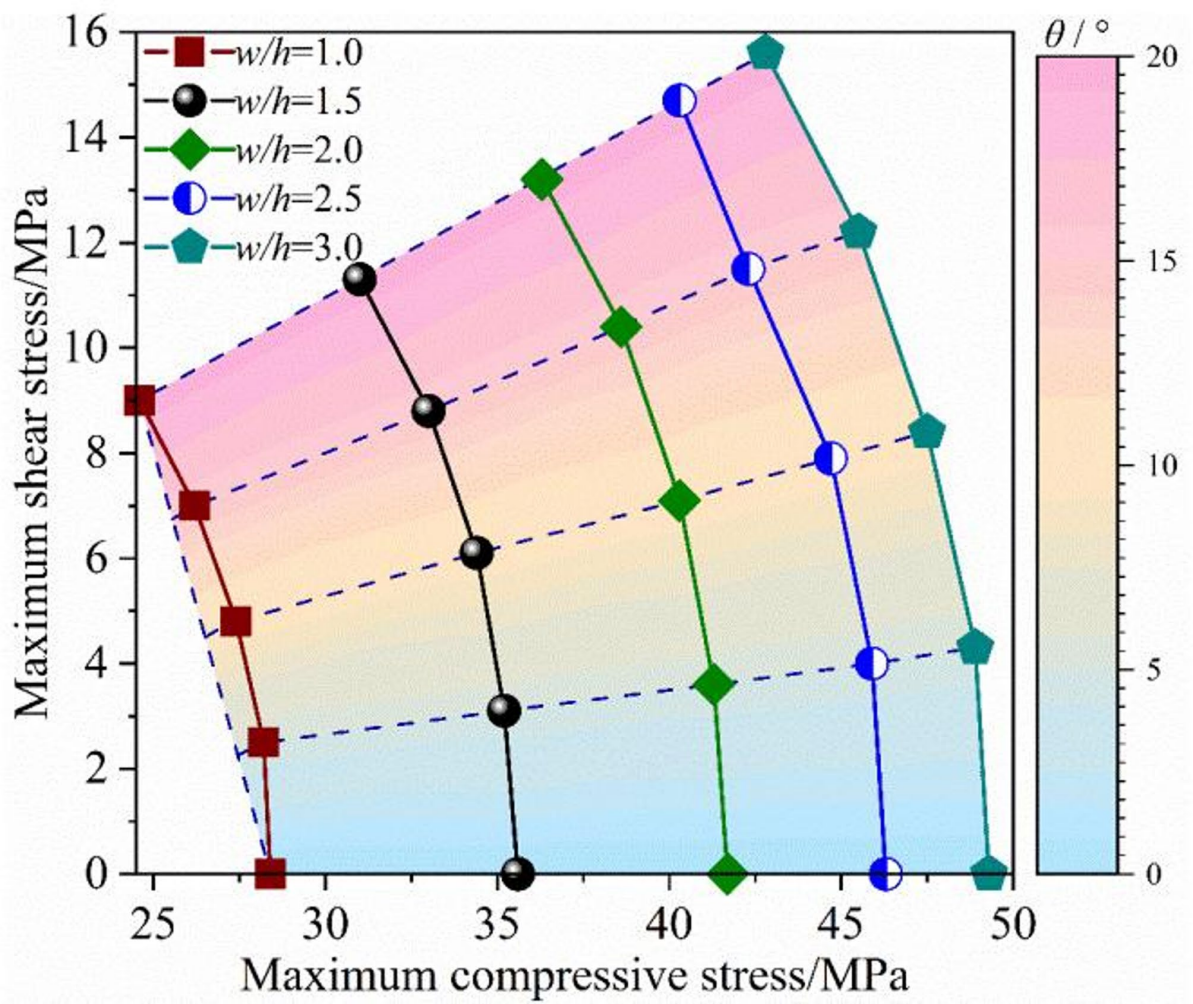
Compressive stress vs. shear stress.

The maximum axial compressive stress and tangential shear stress of the pillar are obtained from the decomposition of Fig. 10. The relationship between the maximum compressive stress and the maximum shear stress is characterized, as shown in Fig. 12. When the width to height ratio is kept constant, the compressive stress component decreases while the shear stress component increases with the increase of orebody dip angle. The relationship between axial compressive stress and shear stress is curvilinear. When the inclination angle remains constant (excluding 0°), an increase in the width-to-height ratio leads to higher compressive stress and shear stress, with a linear relationship observed between the two. As the increase of the width to height ratio, the loci between compression load and shear load outward gradually, which are similar to a ‘spider web’, and the distance between the two tracks gradually decreases. As the width-to-height ratio increases, the maximum compressive stress and maximum shear stress may stabilize and approach constant values rather than continuing to change. Typically, these stresses are influenced by both the dip angle and the width-to-height ratio, exhibiting effects related to both inclination and size.

**Fig. 13:**
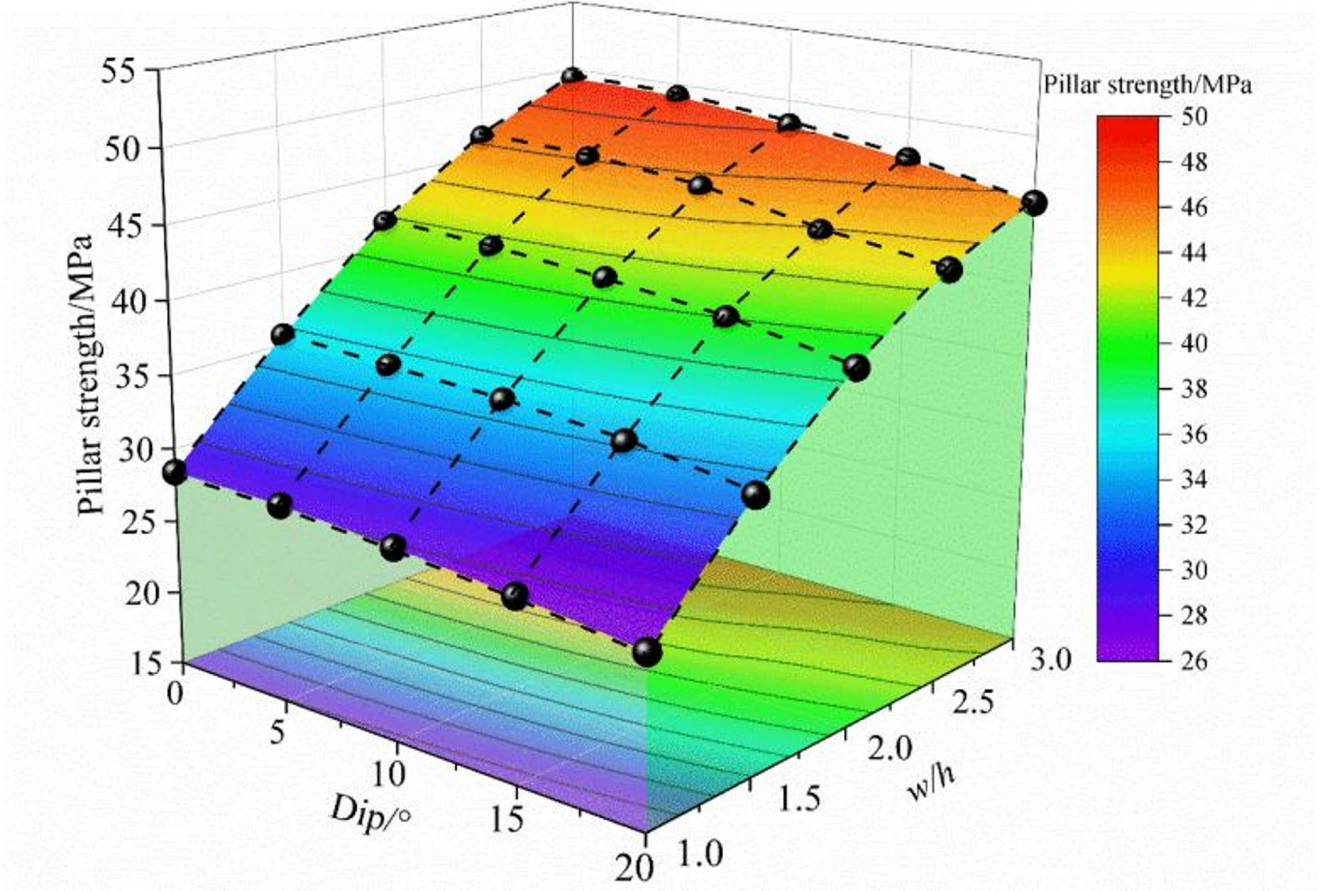
Pillar strength versus both dip and w/h ratio.

Numerical results of inclined pillars strength with different width-height ratios are presented in this paragraph. In terms of the inclined rock pillar, the strength is determined by the magnitude of compressive load and shear load component. The relationship between the maximum compressive and the shear stress is shown in Fig. 11. The strength of the inclined pillar can be obtained, as shown in Fig. 13. When the pillar size remains unchanged, the pillar strength diminishes progressively as the dip angle increases. At a dip angle of 5° for the ore body, the influence of the dip on pillar strength is minimal. However, when the dip angle exceeds 10°, the pillar strength reduces as the dip angle continues to rise. When dip angle is kept constant, pillar strength decreases with the increase of the w/h ratio, pillar strength shows size effect.

#### Stress distribution and failure modes of gently inclined pillars

**Fig. 14:**
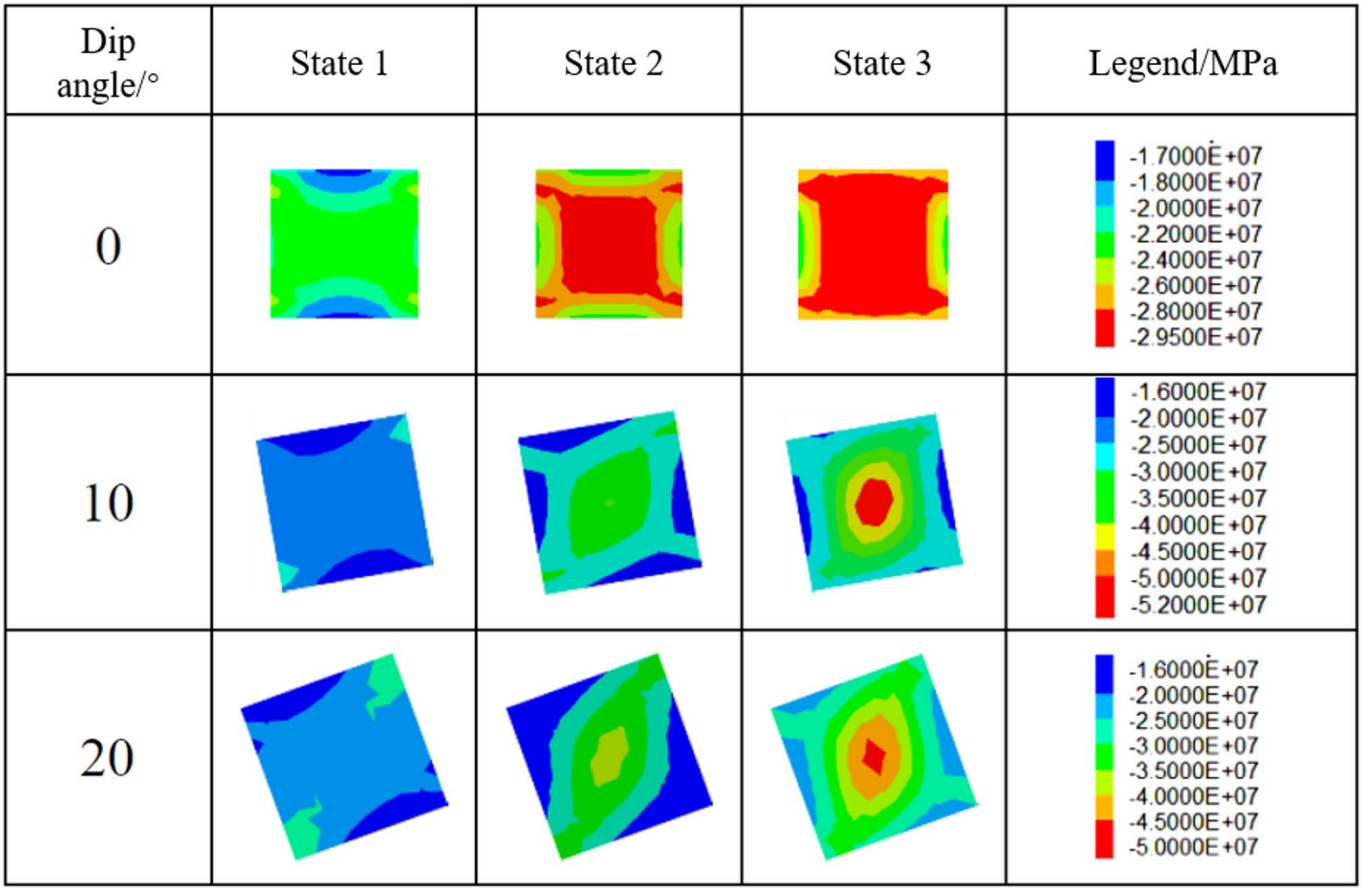
Evolution of maximum principal stress in the pillar from initial loading to failure(W/h = 1).

**Fig. 15:**
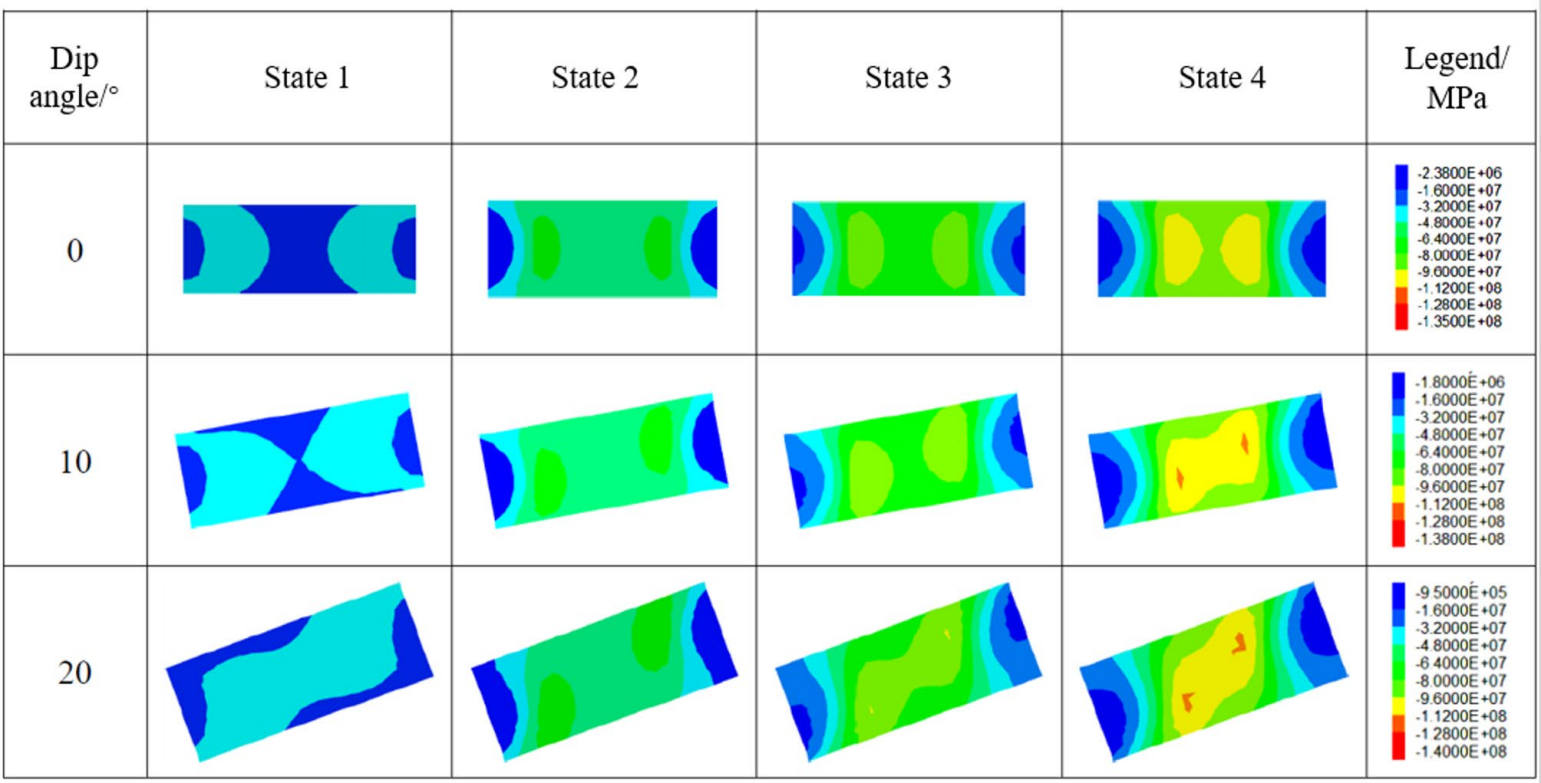
Evolution of maximum principal stress in the pillar from initial loading to failure(W/h = 2.5).

To analyze the influence of pillar inclination on stress state evolution during loading, three dip angles (0°, 10°, and 20°) were investigated. The variation in stress state along the vertical profile of the pillar during analysis is illustrated in Figs. 14 and 15. When the pillar dip angle is 0°, the stress within the pillar remains symmetrically distributed about its central axis as the load increases. For non-zero dip angles, vertical stress intensifies at the corner region between the inclined downward roof and the pillar, where stress concentration initially occurs. With further loading, this concentration progressively migrates toward the pillar center. Concurrently, the supporting pressure diminishes at the corner region between the inclined upward roof and the pillar, leading to stress relaxation that may ultimately evolve into a tensile stress zone. In contrast, stress distributions at the floor-pillar corner regions exhibit opposing trends between upward and downward dip directions compared to those observed in the roof contact zones.

**Fig. 16:**
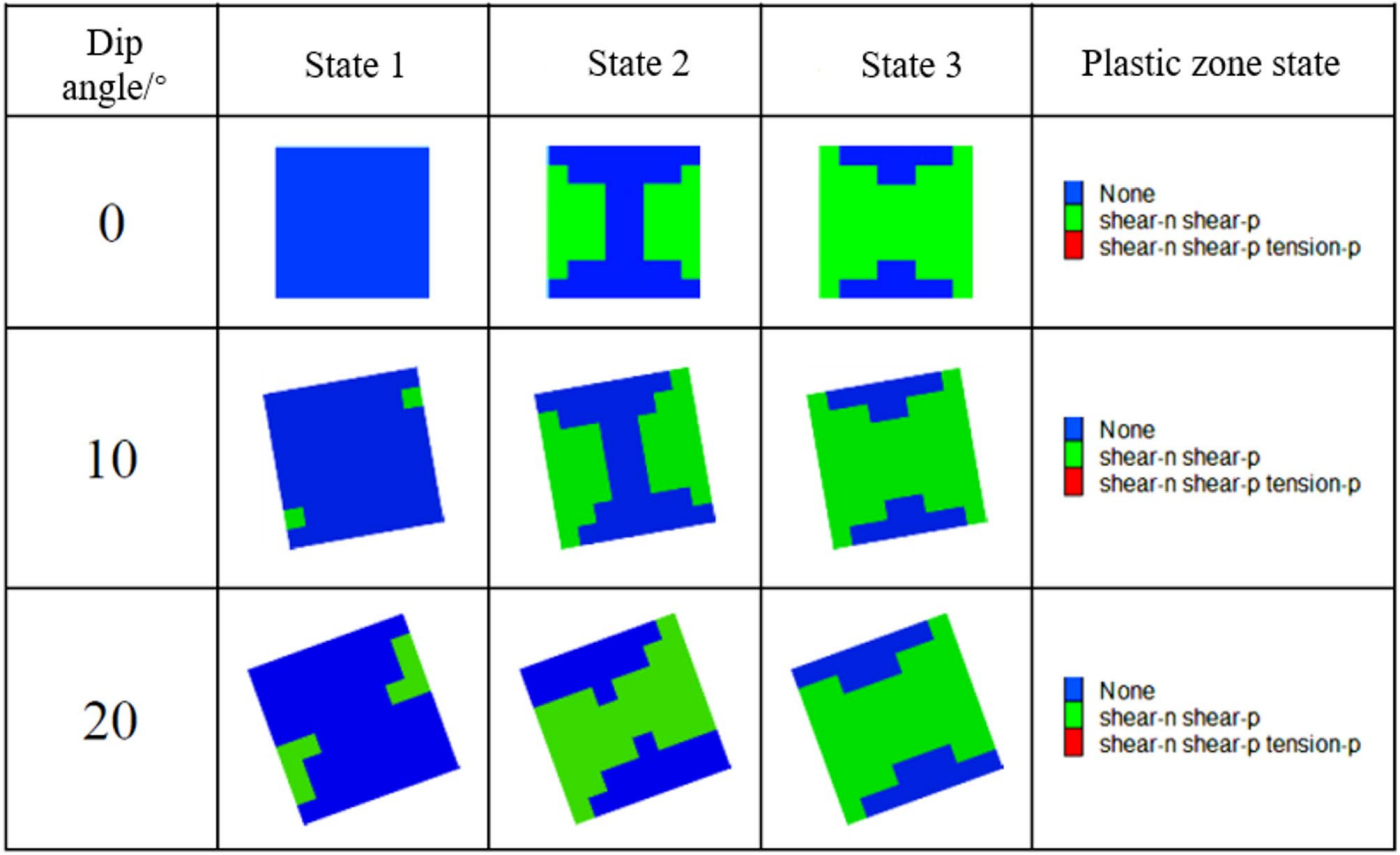
Contour map of plastic zones during pillar failure process (W/h = 1).

**Fig. 17:**
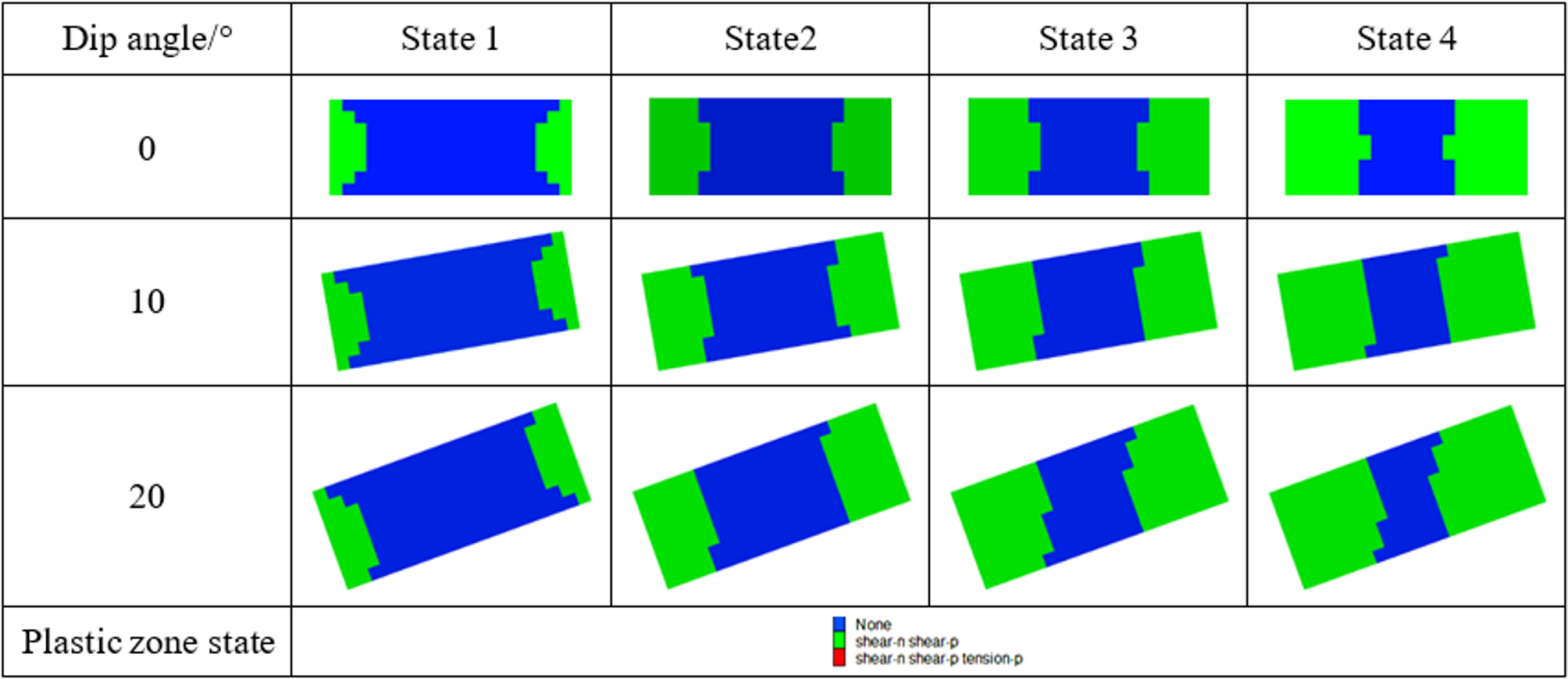
Contour map of plastic zones during pillar failure process (W/h = 2.5).

Figures 16 and 17 illustrate the development process of plastic zones in pillars. When the pillar inclination angle is 0°, the failure exhibits a symmetrical hourglass-shaped pattern under increasing loads until complete destruction occurs, primarily dominated by compressive-shear failure. As the pillar inclination angle increases, the supporting pressure intensifies at the corner region between the downward-inclined roof and the pillar. Stress concentration initially emerges in this area, and when the concentrated stress exceeds the pillar’s bearing capacity, plastic zones first develop in this region, indicating that failure initiates preferentially at this location. Simultaneously, stress redistribution occurs at the corner between the upward-inclined roof and the pillar.

## Discussion and analysis

### Comparative analysis of proposed model and numerical results

**Fig. 18:**
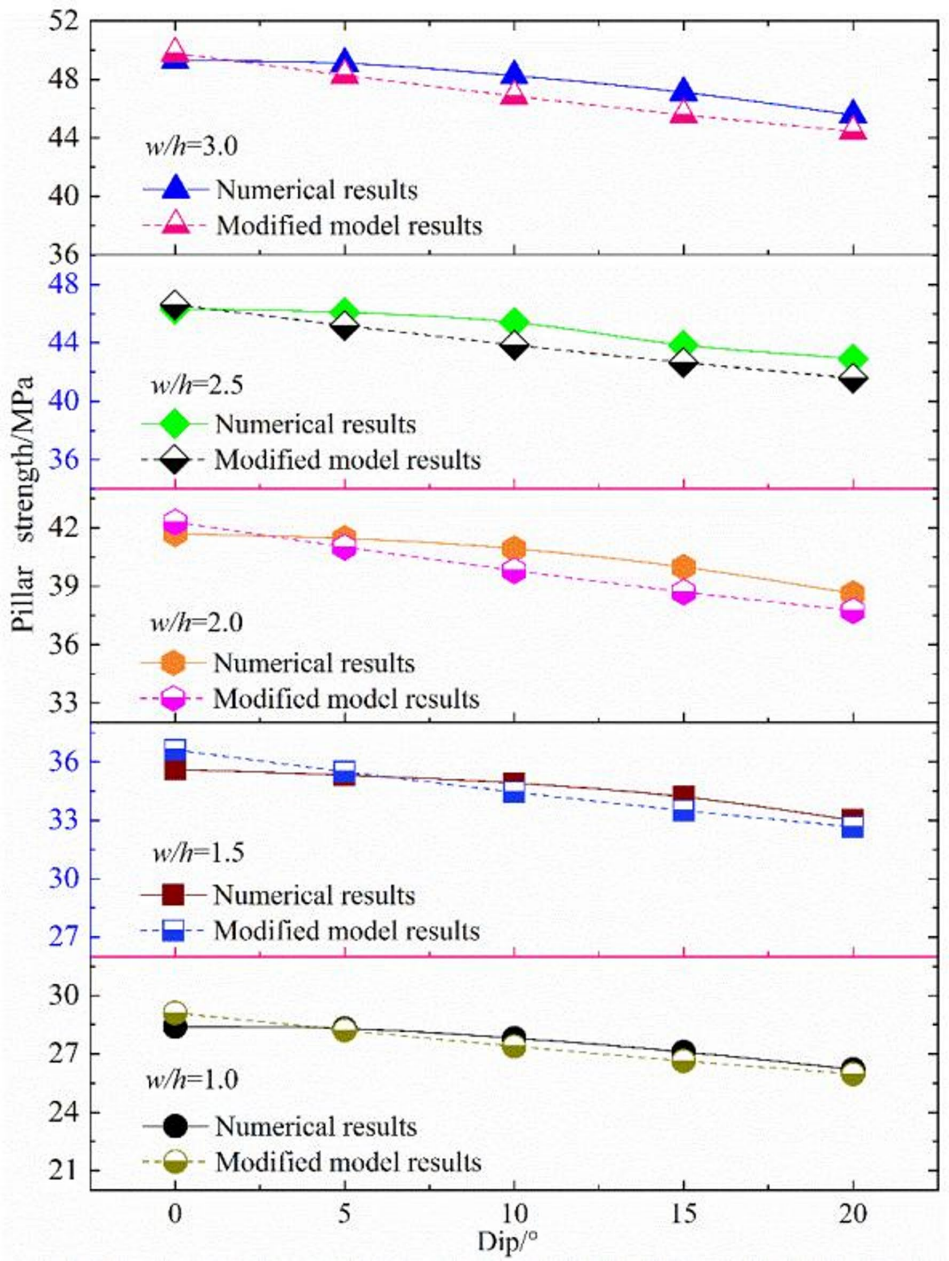
Comparison of rock pillar strength between proposed model and numerical results.

Numerical results were employed to compare with the proposed strength model. Based on the mechanical and geometric parameters of the phosphate rock pillar (Table 2), the pillar strength was determined using the model (Eq. (20)), with the results presented in Fig. 18. Both numerical and model results exhibit that the pillar strength decreases with the increase of the dip angle. The proposed model results are very close to the numerical simulation results, indicating that the model is reasonable. The model provides a way to calculate the strength of gently inclined pillar.

### Factors affecting the strength of inclined pillar

The strength declines with increasing dip angle ranging from 0° to 45°, how to characterize the correlation between the strength and dip angle is a focus issue for scholars. Compression-shear coefficient can characterize the degree of the dip effect on pillar strength. The Eq. (19) is the expression of the compression-shear coefficient of the rock pillar. This coefficient is influenced by the internal friction angle, the dip angle of the ore body, and the in-situ stress ratio. Typically, the dip angle varies between 0° and 90°, though no standardized formula exists for determining the in-situ stress ratio. The most effective method to determine its value is in-situ stress measurement^43^. In the results of in-situ stress measurements around the world, Brown and Hoek^2,3^ summed up the measured range of the in-situ stress. In-situ stress ratios are bounded on the lower side by λ = 0.3, while the upper bound is defined by the expression,

21 $$\lambda =0.3+\frac{{1500}}{H}$$

Where H is the depth below the ground surface in meters; at shallow depth, values of λ vary widely and are frequently much greater than unity. At increasing depth, the variability of the ratio decreases and the upper bound tends towards unity. When H is from 0 to 1000 m, the horizontal stress changes greatly, λ∈[0.3, 3.5]; When H is greater than 1000 m, λ∈[0.3, 1.0]^3^.

**Fig. 19:**
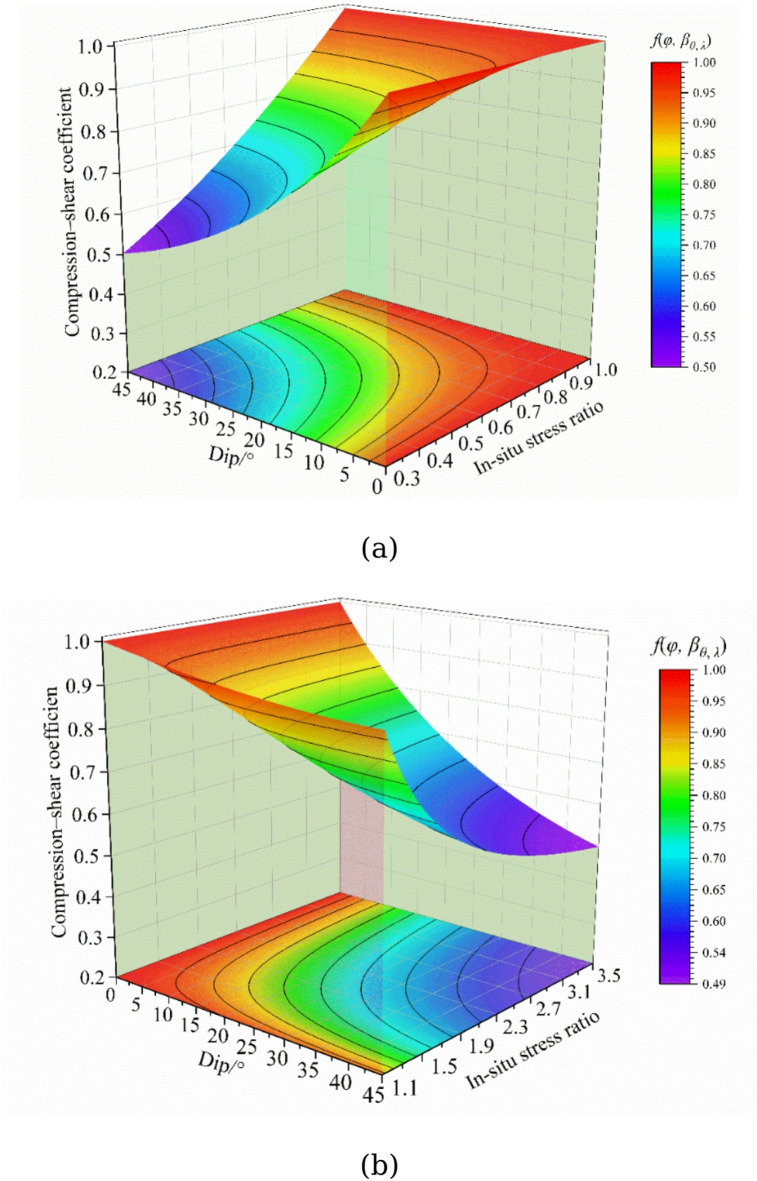
The relationship between compression-shear coefficient of the pillar and both dip angle and in-situ stress ratio; (a) λ∈[0.3, 1.0]; (b) λ∈[1, 3.5].

The relationship between compression-shear coefficient and orebody inclination and in-situ stress ratio is presented in Fig. 19. Figure 19a shows the relationship between the compression-shear coefficient and the ore-body dip angle and the in-situ stress ratio when the in-situ stress ratio is less than 1.0, while the in-situ stress ratio is more than 1.0, the relationship between them is shown in Fig. 19b. When the in-situ stress ratio λ is kept constant, as the increase of dip angle, the compression-shear coefficient of rock pillar decreases gradually under both λ∈[0.3, 1.0] and λ∈[1.0, 3.5]. The difference is that when the in-situ stress ratio is from 0.3 to 1.0, the in-situ stress ratio is the minimum and the compression-shear coefficient gains a minimum value, while the in-situ stress ratio arrives at the maximum value, the minimum compression-shear coefficient will be obtained under λ∈[1.0, 3.5]. Similarly, when a dip angle is kept constant, the compression-shear coefficient increases with the increasing the in-situ stress ratio under λ∈[0.3, 1.0]. While decreases with the increase in the in-situ stress ratio under λ∈[1.0, 3.5].

It should point out that the in-situ stress ratio is considering in the proposed strength model, and the study shows that the in-situ stress ratio has a significant impact on the rock pillar strength^27^. As this paper is mainly concerned with the influence of dip angle on rock pillar strength, detailed and in-depth research of the effect of in-situ stress ratio on rock pillar strength will be the next step.

## Conclusions

In this investigation, the rock strength criterion under combined compression-shear load, built on Mohr-Coulomb strength theory, was used to connect the flat rock pillar strength with the inclined pillar strength. Then the inclined rock pillar strength model was proposed to characterize the dip effect on rock pillar strength. And the numerical simulation method was employed to verify the correctness of the proposed model. The primary conclusions can be drawn as follows.

(1) A strength model including dip angle was proposed to estimate rock pillar strength. Regarding the flat pillar is as a special case of inclined pillar, failure criterion of inclined rock was employed to build the correlation between flat and inclined rock pillar strength. The compression-shear coefficient was employed to link a bridge between flat and inclined rock pillar strength. A strength model was proposed for inclined pillar, which provides a reference for the estimation of inclined pillar strength.

(2) Numerical method was employed to verify the proposed strength model. The magnitude of the compression and shear load components determines the strength of the inclined pillar. The compression load component decreases with increasing dip angle, while the shear load component increases. As a result, pillar strength declines with increasing dip angle. Both numerical model and proposed strength model results show a good agreement under the same parameters, indicating that the proposed model is reasonable and meaningful.

(3) Compression-shear coefficient either is a link between flat pillar strength and inclined pillar strength or can characterize the degree of the dip effect on pillar strength. The internal friction angle, the dip angle of the orebody, and the in-situ stress ratio decide the compression-shear coefficient. Parameters analysis shows that when both dip angle and in-situ stress ratio are not varied, compression-shear coefficient decreases with increasing dip angle ranging from 0° to 45°, which indicates that the pillar strength also declines as increasing dip angle.

## Data Availability

The datasets used and analyzed during the current study are available from the corresponding author on reasonable request.
